# Generation of a Nebulizable CDR-Modified MERS-CoV Neutralizing Human Antibody

**DOI:** 10.3390/ijms20205073

**Published:** 2019-10-12

**Authors:** Sang Il Kim, Sujeong Kim, Jinhee Kim, So Young Chang, Jung Min Shim, Jongwha Jin, Chungsu Lim, Songyi Baek, Ji-Young Min, Wan Beom Park, Myoung-don Oh, Seungtaek Kim, Junho Chung

**Affiliations:** 1 Department of Biochemistry and Molecular Biology, Seoul National University College of Medicine, Seoul 03080, Korea; sangk1128@snu.ac.kr (S.I.K.); sujeong5425@snu.ac.kr (S.K.); 2 Cancer Research Institute, Seoul National University College of Medicine, Seoul 03080, Korea; 3 Department of Biomedical Science, Seoul National University College of Medicine, Seoul 03080, Korea; 4 Respiratory Virus Laboratory, Institut Pasteur Korea, Gyeonggi-do 13488, Korea; jinhee.kim@ip-korea.org (J.K.); soyoung.chang@ip-korea.org (S.Y.C.); ji-young.x.min@gsk.com (J.-Y.M.); 5 Zoonotic Virus Laboratory, Institut Pasteur Korea, Gyeonggi-do 13488, Korea; jungmin.shim@ip-korea.org; 6 New Drug Development Center, 123 Osongsaengmyeng-ro, Cheongju-si, Chungbuk 28160, Korea; jichang011@kbiohealth.kr (J.J.); opern88@kbiohealth.kr (C.L.); bettysongyi1@kbiohealth.kr (S.B.); 7 Department of Internal Medicine, Seoul National University College of Medicine, Seoul 03080, Korea; wbpark1@snu.ac.kr (W.B.P.); mdohmd@snu.ac.kr (M.-d.O.)

**Keywords:** MERS-CoV, aerosol delivery, nebulizer, neutralizing antibody, antibody engineering, pulmonary disease, complementarity-determining regions

## Abstract

Middle East respiratory syndrome coronavirus (MERS-CoV) induces severe aggravating respiratory failure in infected patients, frequently resulting in mechanical ventilation. As limited therapeutic antibody is accumulated in lung tissue following systemic administration, inhalation is newly recognized as an alternative, possibly better, route of therapeutic antibody for pulmonary diseases. The nebulization process, however, generates diverse physiological stresses, and thus, the therapeutic antibody must be resistant to these stresses, remain stable, and form minimal aggregates. We first isolated a MERS-CoV neutralizing antibody that is reactive to the receptor-binding domain (RBD) of spike (S) glycoprotein. To increase stability, we introduced mutations into the complementarity-determining regions (CDRs) of the antibody. In the HCDRs (excluding HCDR3) in this clone, two hydrophobic residues were replaced with Glu, two residues were replaced with Asp, and four residues were replaced with positively charged amino acids. In LCDRs, only two Leu residues were replaced with Val. These modifications successfully generated a clone with significantly greater stability and equivalent reactivity and neutralizing activity following nebulization compared to the original clone. In summary, we generated a MERS-CoV neutralizing human antibody that is reactive to recombinant MERS-CoV S RBD protein for delivery via a pulmonary route by introducing stabilizing mutations into five CDRs.

## 1. Introduction

Middle East respiratory syndrome coronavirus (MERS-CoV) was first identified in Saudi Arabia in 2012 from a patient who suffered acute pneumonia and subsequent renal failure [1]. Since then, the World Health Organization has reported 2254 laboratory-confirmed cases of MERS-CoV infections in 27 different countries around the world, and South Korea has recorded the highest number of cases outside of the Middle East. Despite resilient efforts throughout the scientific and medical communities, no vaccine or antiviral agent for MERS-CoV is currently available.

MERS-CoV is a large (30 kb), enveloped, single-stranded, positive-sense RNA virus. The viral genome encodes four major structural proteins: spike (S), envelope (E), membrane (M), and nucleocapsid (N) proteins [2]. The S glycoprotein is a major envelope protein and interacts with the cellular receptor dipeptidyl peptidase 4 (DPP4) for entry into the host cell [3]. This protein consists of the S1 and S2 subunits. The receptor-binding domain (RBD) within the S1 subunit mediates receptor binding, whereas the S2 subunit facilitates membrane fusion. DPP4 is expressed on a variety of human cells, including fibroblasts, intestinal epithelial cells, and hepatocytes [4], as well as in the lung parenchyma and interstitium [5,6]. MERS-CoV is detected in respiratory secretions and the lower respiratory tract of the infected patients [7,8]. In the most severe cases of MERS-CoV infection, aggravating respiratory failure ultimately results in mechanical ventilation [9]. These observations suggest that the MERS-CoV virus primarily infects the human respiratory tract and replicates within the human airway epithelium [10,11].

Antibodies play a crucial role in the prevention and treatment of viral infection. Polysera taken from recovered patients and vaccinated donors have been used as prophylactic agents for hepatitis B, rabies, and other viral diseases [12,13,14]. Palivizumab (Synagis, Medimmune, Gaithersburg, MD, USA) was approved for the prophylaxis of RSV in 1998, and ibalizumab-uiyk (Trogarzo, TailMed Biologics, Taiwan) became clinically available in 2018 for the treatment of human immunodeficiency virus type 1 (HIV-1) infection in treatment-experienced adults with multi-drug-resistant HIV-1 and failure to respond to the current antiretroviral regimen.

In response to the ongoing epidemic, several groups have developed anti-MERS-CoV neutralizing monoclonal or polyclonal antibodies that target RBD [15,16]. These antibodies were generated from B cells derived from convalescent patients, nonimmune human antibody phage-display libraries, fully humanized mice, transchromosomic bovines, or hybridomas from mice that were immunized with MERS-CoV S. These antibodies potently inhibit RBD binding to the DPP4 receptor [17,18,19,20,21,22,23]. Furthermore, therapeutic effects of RBD-specific neutralizing antibodies were evaluated in several animal models, including Ad5/hDPP4-trasduced mice, humanized DPP4 mice, and hDPP4-transgenic mice as well as hDPP4-knock-in mice, rabbits, and rhesus monkeys [17,21,24,25,26,27,28,29,30].

All MERS-CoV neutralizing antibodies were developed for intravenous (i.v.) delivery; however, recent reports indicate that the amount of antibody delivered to lung tissue is often quite limited following systemic delivery [31,32]. In cynomolgus monkeys, bronchoalveolar lavage fluid contained dose-proportional concentrations of systemically administrated antibody, and these concentrations were approximately 500-fold less than those in plasma [31]. Therefore, delivery of therapeutic antibody to lung tissues via inhalation has garnered considerable interest. Following delivery via the airway, cetuximab, an anti-epidermal growth factor receptor (EGFR) antibody, accumulated in normal and cancerous tissues in the lung at a concentration that was twice that achieved after i.v. delivery [33]. In addition, recent studies showed that Fc fusion proteins and nanobodies are also efficiently delivered via the pulmonary route [34,35,36,37]. Therefore, MERS-CoV neutralizing antibody may also accumulate at higher concentrations following delivery via a pulmonary route, suggesting higher efficacy. In order for this pulmonary delivery to be successful, the antibody must be sufficiently stable to resist denaturation during the process of nebulization.

In this study, we generated a MERS-CoV neutralizing antibody for delivery via nebulization. We constructed a phage-display library from two convalescent MERS-CoV-infected patients and successfully isolated nine MERS-CoV RBD-specific neutralizing mAbs. After nebulization, these antibodies showed significant aggregation and reduced reactivity to recombinant S glycoprotein. We therefore reduced the number of hydrophobic residues and introduced solubilizing mutations within the complementarity-determining regions (CDRs), generating an antibody that is resistant to aggregation during nebulization and retains its MERS-CoV neutralizing activity.

## 2. Results

### 2.1. Generation of Antibodies Reactive to Recombinant MERS-CoV RBD Protein From Patients

We generated human single-chain variable fragment (scFv) phage-display libraries using peripheral blood mononuclear cells (PBMCs) isolated from two MERS-CoV-infected convalescent patients. One patient (P014) was considered to be the super spreader, and the other patient (P002) was the wife of the index patient in the previous report [38]. The complexity of the libraries exceeded 3.6 × 10^9^ and 1.9 × 10^9^ colony-forming units for patients P002 and P014, respectively. After the third and fourth rounds of biopanning against recombinant MERS-CoV S RBD protein, the scFv clones were retrieved in a high-throughput manner as described previously [39]. Briefly, 1800 microcolonies formed on the TR chip, and of these, 542 clones with unique *V_H_* and *V_Κ_*/*V_λ_* were identified. In these clones, 44 unique HCDR3 sequences were identified. We selected 44 clones encoding unique HCDR3 sequences and rescued phages for phage enzyme-linked immunosorbent assay (ELISA) analysis. A total of 36 unique scFv clones were highly reactive to recombinant MERS-CoV S RBD protein (data not shown). These clones were prepared as scFv fused with human Fc (scFv-hFc) using a eukaryotic expression vector and HEK293F cells. A human anti-MERS-CoV neutralizing mAb reported previously, m336, was also prepared in this same form for use as a positive control [40].

### 2.2. Selection of MERS-CoV Neutralizing Antibodies

We performed a microneutralization assay to test the neutralizing activity of the 36 identified scFv clones against MERS-CoV (MERS-CoV/KOR/KNIH/002_05_2015). Among these, scFV clones 10, 15, 20, C-8, 34, 42, 46, 47, and 48 potently inhibited MERS-CoV replication, with half-maximal inhibitory concentration (IC_50_) values ranging from 2.40 to 9.61 μg/mL (Table S1).

Next, we tested the stability of these clones during nebulization. We nebulized the fusion proteins at a concentration of 100 μg/mL in phosphate-buffered saline (PBS) using a vibrating mesh nebulizer and then collected the aerosol. All the collected samples showed clearly visible aggregation (data not shown). After centrifugation to remove the aggregated material, we repeated the ELISA analysis and compared the reactivity of pre- and post-nebulized scFv-hFc. All nine clones showed significantly reduced reactivity against recombinant S glycoprotein after nebulization (Figure S1).

We selected the clones C-8 and 48, as these antibodies exhibited the lowest IC_50_ values among the antibodies derived from patients P002 and P014, respectively. Before performing further studies, we studied the mechanism underlying inhibition of viral infection on cells. The antibodies were mixed with recombinant S glycoprotein and added to hDPP4-expressing Huh-7 cells. Both C-8 and 48 scFv-hFc nearly completely blocked binding of recombinant S glycoprotein to cells at equimolar concentration of 100 nM (Figure S2), indicating that the antibodies block the initial interaction of the virus with cells.

### 2.3. Modification of CDR Residues to Enhance Antibody Stability

**Figure 1:**
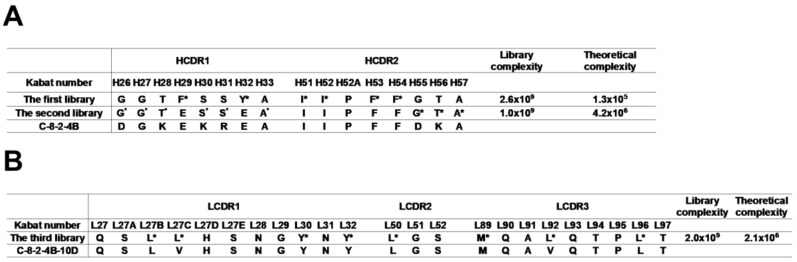
Sequential randomization of CDR residues of the C-8 clone. (**A**) In the first randomized library, six hydrophobic amino acid residues (asterisks) in HCDR1 and HCDR2 were targeted. The second library was prepared in the C-8-2 clone by randomizing nine amino acid residues (asterisks) that were not randomized in the first randomized library. (**B**) Eight amino acid residues (asterisks) in LCDRs of the C-8-2-4B clone selected from the second library were randomized in the third randomized library.

To enhance the stability of the C-8 and 48 clones, we sought to introduce mutations in CDRs, except for heavy chain CDR3 (HCDR3), for replacement of hydrophobic residues with hydrophilic residues. We defined CDRs according to the International Immunogenetics Information System (IMGT) and targeted Phe, Ile, Leu, Val, Met, Trp, and Tyr which were defined as hydrophobic amino acids in previous reports [41,42]. For the C-8 clone, the F29, Y32, I51, I52, F53, and F54 hydrophobic residues in HCDR1 and HCDR2 were selected for randomization (Figure 1A). These six residues were designed to encode the wild-type amino acid, Asp, Glu, or redundant amino acids depending on the degenerate codon in the first scFv phage-display library (Table S2). We preferred negatively charged amino acids to positively charged amino acids as lowering the isoelectric point of an antibody may reduce the non-specific *in vivo* clearance [43]. The randomized scFv phage-display library had a complexity of 2.6 × 10^9^ colony-forming units, which exceeded the theoretical complexity of 1.3 × 10^5^ on the nucleotide level. After two rounds of biopanning on recombinant MERS-CoV S RBD protein, we randomly rescued phage clones and performed phage ELISA. Eleven scFv clones showed reactivity to recombinant MERS-CoV S RBD protein similar to or higher than that of the original C-8 clone. The C-8-2 clone harbored F29E and Y32E replacements, while the other ten clones had only one residue replaced with either Asp, Glu, or redundant amino acids, depending on the degenerate codon. To test the stability of the C-8-2 clone during nebulization, a scFv-hFc fusion protein was prepared and subjected to ELISA following nebulization. The reactivity of C-8-2 scFv-hFc to recombinant S glycoprotein was much less affected by nebulization than that of C-8 scFv-hFc; however, the reactivity of the C-8-2 clone was somewhat reduced compared with that of the C-8 clone (Figure S3A).

In a parallel experiment using clone 48, we prepared a randomized scFv phage-display library and selected seven clones. None of the clones were successfully expressed in the scFv-hFc format (less than 300 μg/L), preventing us from conducting further studies on clone 48 (data not shown).

To achieve further stabilization and affinity maturation, we generated a second scFv phage-display library using the same strategy to randomize nine residues in HCDR1 and HCDR2 of the C-8-2 clone to introduce more negatively charged residues (Figure 1A, Table S2). The proline at H52A was excluded from the randomization, as proline frequently forms a unique structure essential for antibody reactivity [44]. The second randomized scFv phage-display library had a complexity of 1.0 × 10^9^ colony-forming units, which exceeded the theoretical complexity of 4.2 × 10^6^ on the nucleotide level. After the second round of biopanning on recombinant MERS-CoV S RBD protein, we selected 12 clones that displayed greater reactivity to recombinant MERS-CoV S RBD protein than the C-8-2 clone in phage ELISA analysis. Clone C-8-2-4B contained replacement at six residues (G26D, T28K, S30K, S31R, G55D, and T56K; Figure 1A) and showed the highest intrinsic solubility score [45] among the 12 tested clones. Interestingly, only two residues were replaced with Asp, and four residues were replaced with positively charged amino acids, as allowed by the degenerate codons (Figure 1A). We then prepared a C-8-2-4B scFv-hFc fusion protein using a eukaryotic expression system. After nebulization, the reactivity of C-8-2-4B scFv-hFc to recombinant S glycoprotein was less affected than either C-8 or C-8-2 scFv-hFc (Figure S3A,B). In addition, the reactivity of C-8-2-4B scFv-hFc was enhanced compared to that of C-8-2 scFv-hFc and comparable to that of C-8 scFv-hFc.

Next, we prepared C-8 and C-8-2-4B IgG_1_ using a eukaryotic expression system and compared the reactivity of these immunoglobulins to recombinant S glycoprotein before and after nebulization. As expected, the reactivity of C-8-2-4B IgG_1_ was better retained following nebulization than that of C-8 IgG_1_ (Figure S3C). We also tested whether C-8-2-4B IgG_1_ effectively blocked the interaction between recombinant S glycoprotein and hDPP4-expressing Huh-7 cells after nebulization. In flow cytometry analysis, we found that C-8-2-4B IgG_1_ almost completely blocked the binding of recombinant S glycoprotein to hDPP4-expressing cells following nebulization, while C-8 IgG_1_ failed to block this interaction after nebulization (Figure S3D).

**Figure 2:**
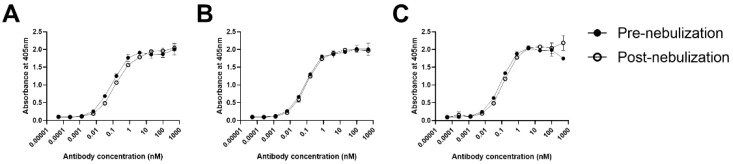
Reactivity of anti-MERS-CoV IgG_1_ antibodies before and after nebulization. Following nebulization at a concentration of 1 mg/mL, aerosol was collected and subjected to ELISA. Recombinant S glycoprotein-coated microtiter plates were incubated with pre-nebulized and post-nebulized C-8 IgG_1_ (**A**), C-8-2-4B-10D IgG_1_ (**B**), and m336 (**C**). HRP-conjugated anti-human IgG antibody was used as the probe, and ABTS was used as the substrate. All experiments were performed in duplicate, and the data indicate mean ± SD.

As C-8-2-4B IgG_1_ showed a somewhat reduced reactivity after nebulization, we sought to confer additional stability by randomizing eight hydrophobic residues in LCDRs using the same randomization scheme. We achieved 2.0 × 10^9^ colony-forming units in the third randomized scFv phage-display library, exceeding the theoretical complexity of 2.1 × 10^6^ (Figure 1B). After two rounds of biopanning on recombinant MERS-CoV S RBD protein, we selected clones in a phage ELISA with reactivity similar to or greater than that of C-8-2-4B. Sanger sequencing revealed that a single clone was repetitively selected. The selected clone, C-8-2-4B-10D, harbored replacements at L27C and L92V with valine (Figure 1B). We prepared C-8-2-4B-10D IgG_1_ using a eukaryotic expression system and analyzed the characteristics using ELISA, size-exclusion high-performance liquid chromatography (SE-HPLC), dynamic light scattering (DLS), and plaque reduction neutralization tests (PRNT_50_). ELISA revealed a noticeable decline in reactivity to recombinant S glycoprotein by C-8 IgG_1_ and m336 IgG_1_ after nebulization; yet, the change in reactivity of C-8-2-4B-10D IgG_1_ after nebulization was negligible (Figure 2).

**Figure 3:**
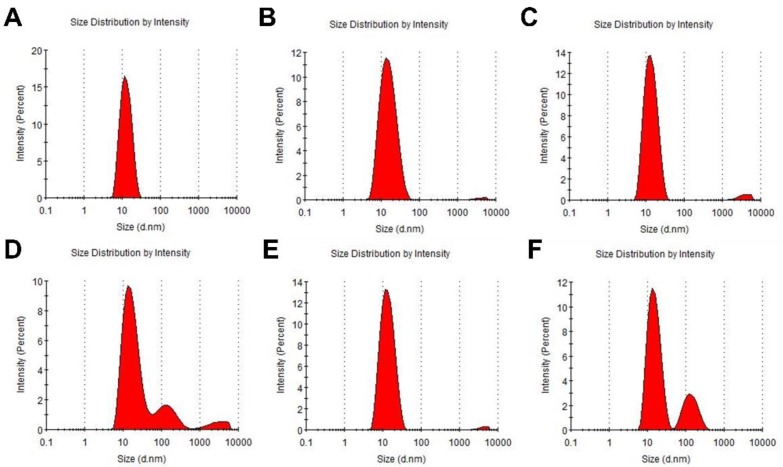
DLS analysis. To evaluate the size distribution profile of pre-nebulized C-8 (**A**), pre-nebulized C-8-2-4B-10D (**B**), pre-nebulized m336 (**C**), post-nebulized C-8 (**D**), post-nebulized C-8-2-4B-10D (**E**), and post-nebulized m336 IgG_1_ (**F**) antibodies, DLS was performed using 633-nm/4-mW laser at a 173° detection angle. PBS was used as the reference solvent, and the results were evaluated with Zetasizer software 7.02. All experiments were performed in triplicate, and representative results are shown for each antibody.

In SE-HPLC analysis, high-molecular weight aggregates were detected in post-nebulization samples of C-8 and m336 IgG_1_; however, no aggregate was found in post-nebulized samples of C-8-2-4B-10D IgG_1_ (Table 1, Figure S4). In accordance with these SE-HPLC data, DLS analysis showed that the nebulization process converted 21.6% and 22.5% of C-8 and m336 IgG_1_, respectively, into high-molecular-weight aggregates, while nebulization resulted in <1% aggregates for C-8-2-4B-10D IgG_1_. (Table 1, Figure 3).

### 2.4. Neutralizing Potency After Nebulization

**Figure 4:**
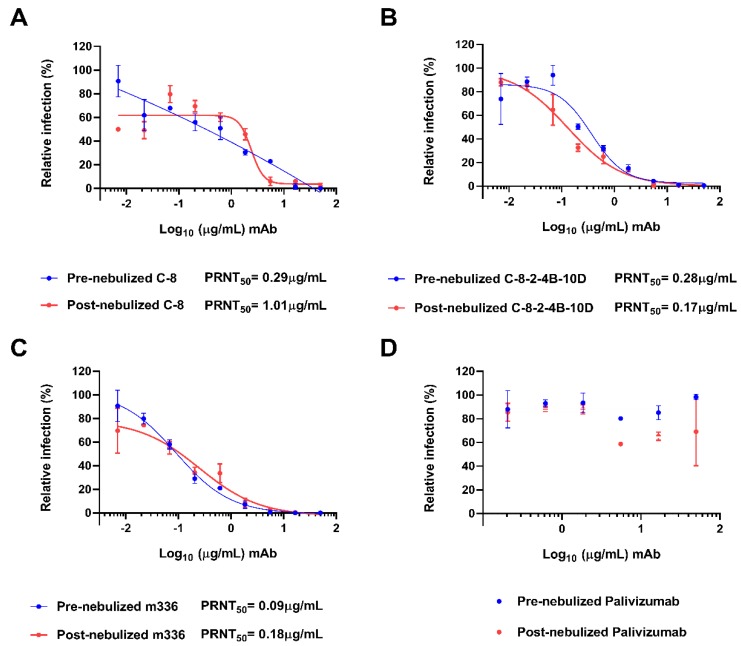
Neutralization of MERS-CoV by pre- and post-nebulized IgG_1_. Culture media containing 100 PFU MERS-CoV was mixed with equal volume of serially diluted C-8 IgG_1_ (**A**), C-8-2-4B-10D IgG_1_ (**B**), m336 IgG_1_ (**C**), and palivizumab (**D**). After incubation for 1 h, the mixture was added to Vero cells. After 2 days, the plaques were counted. The inhibition of virus infection was plotted as a function of IgG_1_ antibody concentration, and PRNT_50_ values were calculated by GraphPad Prism 6. All experiments were performed in quadruplicate, and the data indicate mean ± SD.

The neutralizing activities of pre- and post-nebulized C-8 and C-8-2-4B-10D IgG_1_ were evaluated in PRNT_50_ using the live MERS-CoV (MERS-CoV/KOR/KNIH/002_05_2015). Antibodies were mixed with live MERS-CoV, and then the antibody-virus mixture was allowed to infect Vero cells. C-8 and C-8-2-4B-10D IgG_1_ exhibited effective inhibitory activity against MERS-CoV, with IC_50_ values of 0.29 and 0.28 μg/mL, respectively. After nebulization, C-8-2-4B-10D showed an IC_50_ value similar to that of pre-nebulized IgG_1_, but the IC_50_ value of C-8 was dramatically increased following nebulization (Figure 4).

## 3. Discussion

Pulmonary delivery can be an efficient drug delivery route to the lung parenchyma, and such delivery can sometimes exceed the efficiency of systemic injection [33]. To deliver drug via the airways, an aerosol that contains the drugs is generated by a nebulizer [46]; however, the physical stress of nebulization often causes protein instability by affecting the integrity of the molecular structure, frequently resulting in fragmentation and aggregation [47]. Aggregation of therapeutic proteins is a major concern, as it contributes to immunogenicity, which frequently causes adverse events, such as decreased drug efficacy, infusion reactions, cytokine release syndrome, or anaphylaxis [48]. A vibrating mesh nebulizer, which was designed for protein delivery, generates limited variation on the temperature, concentration, and surface tension, and its effect on the stability of the protein is the least among nebulizers [49]. This type of nebulizer also produces uniform sized particles and flow rates, which are also beneficial in maintaining the stability of biological products [37].

To reduce the immunogenicity of therapeutic protein, maintaining the stability of the native protein conformation as well as minimal (or no) formation of high-molecular weight species are crucial [50]. Therefore, engineering of a protein to render it more stable for pulmonary delivery is important. In a recent study, a trivalent nanobody against RSV F protein (ALX-O171) was successfully delivered directly into the lungs by nebulization and neutralized RSV in newborn lambs [46]. In this case, the framework 2 region of the nanobody contained more hydrophilic residues that are not observed in human *V_H_* domains and thereby increasing the stability of the nanobody [37]. In this study, we focused on CDRs, as the sequences of the CDR loops are closely related to the folding stability of antibodies [51,52]. CDR loops frequently possess hydrophobic residues to facilitate high binding affinity; however, solvent-exposed hydrophobic residues also impact antibody stability and aggregation [53,54,55]. To increase solubility and counterbalance the impact of the hydrophobic residues required for antibody binding, solubilizing residues can be introduced either at the edges of the CDR loops or within the CDR [56,57]. Furthermore, negatively charged substitution mutations within CDRs can be used to prevent aggregation [58]. In our study, we employed both strategies and reduced the number of hydrophobic residues and increased the number of charged residues in the CDRs, resulting in successful enhancement of the stability of a MERS-CoV neutralizing antibody. The final optimized antibody, C-8-2-4B-10D, showed very limited protein aggregation after nebulization and its biological potency was well maintained after such delivery. Further, we expect that formulation with surfactants such as polysorbate may prevent aggregation of this antibody during nebulization. 

To test whether the C-8-2-4B-10D antibody provides better efficacy when delivered via a pulmonary route than via systemic injection, an animal model with progressive pulmonary failure is essential. In the case of hDPP4-transgenic mouse models, the infected mice exhibited central nervous system and multi-organ failure but no severe pulmonary symptoms [59,60,61]. Recently, hDPP4-knock-in mice were developed and showed progressive pulmonary manifestations when infected with a mouse-adapted strain [62]. Thus, in future studies, we will test the efficacy of C-8-2-4B-10D delivered via pulmonary route in these hDPP4-knock-in mice.

## 4. Materials and Methods

### 4.1. Ethics Statement

The study that provided the human samples was approved by the Institutional Ethics Review Board of Seoul National University Hospital (IRB approval number: 1602-100-742), and written informed consent was obtained from all participants.

### 4.2. Construction of A Human scFv Phage-Display Library and Three Randomization Libraries

PBMCs were isolated from two MERS-CoV-infected convalescent patients using a Ficoll-Paque density gradient medium (GE Healthcare, Pittsburgh, PA, USA) as described previously [63]. The PBMCs were subjected to total RNA isolation using the TRI Reagent (Invitrogen, Carlsbad, CA, USA) following the manufacturer’s instructions. The RNA was used to synthesize cDNA using Superscript III First-Strand Synthesis system (Invitrogen) with oligo(dT) primers according to the manufacturer’s instructions. Using the cDNA as a template, the genes encoding the variable regions of heavy and light chains (*V_H_* and *V_Κ_/V_λ_*) were amplified and used for the construction of a human scFv phage-display libraries as described previously [64,65].

For the construction of the first randomization library, a set of degenerate Ultramer DNA oligonucleotides (Integrated DNA Technologies, Coralville, IA, USA) encoding residues from H1 to H65 of clone C-8 (V_HN1_) was chemically synthesized to contain either a codon encoding the wild-type amino acid or a GAK degenerate codon at the H29, H32, H51, H52, H53, and H54 residues (Table S2). Then, the gene fragment (V_HC_) encoding residues from H58 to H113 of clone C-8 was amplified by PCR using primer set 1 (Table S3) in a T100 Thermal Cycler (Bio-Rad, Carlsbad, CA, USA). The PCR conditions were as follows: preliminary denaturation at 94 °C for 5 min, followed by 25 cycles of 15 s at 94 °C, 15 s at 56 °C and 90 s at 72 °C. A final extension was then conducted for 10 min at 72 °C. After electrophoresis on a 1% agarose gel, the PCR products were purified using QIAquick gel extraction kit (Qiagen Inc., Valencia, CA, USA) according to the manufacturer’s instructions. The purified V_HN1_ and V_HC_ gene fragments were mixed at a concentration of 100 ng and subjected to linker PCR using primer set 2 (Table S3) in a T100 Thermal Cycler to yield the V_H1_ fragment. The PCR conditions were as follows: preliminary denaturation at 94 °C for 5 min, followed by 25 cycles of 15 s at 94 °C, 15 s at 56 °C and 120 s at 72 °C. The reaction was ended with an extension step for 10 min at 72 °C. The gene fragment encoding *V_L_* (V_L1_) of clone C-8 was amplified by PCR using primer set 3 (Table S3) with the same PCR conditions described above for amplification of V_HC_. Then, the V_H1_ and V_L1_ fragments were subjected to electrophoresis on a 1% agarose gel, and excised bands were purified using the QIAquick gel extraction kit. The purified V_H1_ and V_L1_ fragments were used for the synthesis of the scFv gene (scFv_1_) using PCR as described previously [64]. The amplified scFv_1_ fragment was purified and cloned into the phagemid vector as described [64,65].

For the construction of the second randomization library, a set of degenerate Ultramer DNA oligonucleotides encoding residues from H1 to H65 of clone C-8-2 (V_HN2_) was chemically synthesized to contain either a codon encoding the wild-type amino acid or a GAK degenerate codon at the H26 to H33 (HCDR1) and H51 to H57 (HCDR2) residues (Table S2), excluding the previously randomized residues. The V_HN2_ and V_HC_ gene fragments were mixed at equal ratios at 100 ng and subjected to linker PCR using primer set 2 (Table S3) in a T100 Thermal Cycler to yield the V_H2_ gene fragment as described above. The V_H2_ gene fragment was purified as described above and subjected to linker PCR with V_L1_ fragments to yield the scFv_2_ gene fragment, which was cloned into the phagemid vector as described above.

For the construction of the third randomization library, two sets of degenerate Ultramer DNA oligonucleotides with a length of 200 nucleotides were chemically synthesized. One set encoded from L1 to L61 residues of clone C-8 (V_LN_), while the other one encoded from L56 to L107 of clone C-8 (V_LC_). These degenerate oligonucleotides contained either a codon encoding the wild-type amino acid or a GAK degenerate codon at L27B, L27C, L30, L32, L50, L89, L92, and L96 residues (Table S2). The V_LN_ and V_LC_ gene fragments (100 ng each) were subjected to a linker PCR using primer set 3 (Table S3) in a T100 Thermal Cycler to produce the VL_2_ gene fragment using the same PCR conditions as described above for the amplification of the V_H1_ gene fragment. The gene fragment encoding V_H_ of C-8-2-4B (VH_3_) was amplified by PCR using primer set 2 (Table S3) using the same PCR conditions used for the amplification of the V_HC_ gene fragment as described above. After purification, VL_2_ and VH_3_ gene fragments were used to produce the scFv_3_ gene fragment, which was cloned into the phagemid vector as described above.

### 4.3. Biopanning

The human scFv phage-display libraries were subjected to four rounds of biopanning against recombinant MERS-CoV S RBD protein (Sino Biological Inc., Beijing, China) as described previously [66]. Briefly, the scFv phage-display libraries (~10^11^ phage) were added to 3 μg of the recombinant MERS-CoV S RBD protein conjugated to 5.0 × 10^6^ magnetic beads (Dynabeads M-270 epoxy, Invitrogen) and incubated with rotation for 2 h at 37 °C. The beads were washed once with 500 μL of 0.05% (*v*/*v*) Tween-20 (Sigma-Aldrich, St. Louis, MO, USA) in PBS (PBST) during the first round of biopanning. The number of washes was increased to three for the other rounds. Phages bound to beads were eluted, neutralized, allowed to infect *E. coli* ER2738 (New England Biolabs, Ipswich, MA, USA), and rescued as described previously [66].

The first randomized scFv library was subjected to two rounds of biopanning against recombinant MERS-CoV S RBD protein. The scFv phage-display library (~10^11^ phage) was added to 1.5 μg of the recombinant MERS-CoV S RBD protein conjugated to 2.5 × 10^6^ magnetic beads and incubated with rotation for 2 h at 37 °C. The beads were washed once with 500 μL of 0.5% PBST and three times with 500 μL of 0.5% PBST during the first and second rounds of biopanning, respectively. After each round of washing, bound phages were eluted and rescued as described above.

For first round of biopanning for the second and third randomized scFv libraries, the scFv phage-display libraries (~10^11^ phage) were added to 1.5 μg of the recombinant MERS-CoV S RBD protein conjugated to 2.5 × 10^6^ magnetic beads and incubated with rotation for 2 h at 37 °C. After washing three times with 500 μL of 0.5% PBST, bound phages were eluted and rescued as described above.

Before the second round of biopanning of the second and third randomized scFv libraries, 10 μg of recombinant MERS-CoV S RBD protein was conjugated to 200 μg of non-magnetic beads (Nacalai, San Diego, CA, USA) following the manufacturer’s instructions. Then, the scFv phage-display libraries (~10^11^ phage) were added to 1.5 μg of recombinant MERS-CoV S RBD protein conjugated to 2.5 × 10^6^ magnetic beads and incubated on a rotator for 2 h at 37 °C. After washing three times with 500 μL of 0.5% PBST, magnetic beads were resuspended in 100 μL of PBS and transferred to a microtube (microTUBE AFA Fiber Pre-Slit Snap-Cap, 520045, Covaris, Woburn, MA, USA) along with the recombinant MERS-COV S RBD protein-conjugated non-magnetic beads resuspended in 30 μL of PBS at a concentration of 0.33 μg/mL. Then, these bead mixtures were subjected to an ultrasound washing step using an ultrasonicator (M220, Covaris) with the following conditions: duty factor (DF) 20%, peak incident power (PIP) 12.5 W, cycles/burst 50, 20 min, and 24 °C. After ultrasonication, magnetic beads were transferred to 1.5-mL microcentrifuge tube and washed three times with 0.5% PBST. Then, the bound phages were eluted and rescued as described above.

### 4.4. High-Throughput Retrieval of scFv Clones and Phage ELISA

After the fourth round of biopanning of human scFv phage-display libraries, the plasmid DNA was obtained from overnight cultures of *E. coli* cells and subjected to high-throughput retrieval of scFv clones by TrueRepertoire analysis as described previously (Celemics, Seoul, Korea) [39].

To select reactive clones to recombinant MERS-CoV S RBD protein, the scFv genes obtained from TrueRepertoire were cloned into the pComb3XSS vector [64] and used to transform *E. coli* ER2738 cells. After overnight culture, the phages were rescued from individual colonies using the M13K07 helper phage and subjected to phage ELISA as described previously [64]. Microtiter plates (Costar, Cambridge, MA, USA) were coated with 100 ng of recombinant MERS-CoV S RBD protein in coating buffer (0.1 M sodium bicarbonate, pH 8.6) at 4 °C overnight. The wells were blocked with 3% (*w*/*v*) bovine serum albumin (BSA; Thermo Scientific, Waltham, MA, USA) dissolved in PBS for 1 h at 37 °C, and culture supernatant containing scFv-displayed phages that were rescued from individual colonies were added into each well. After incubation for 2 h at 37 °C, the microtiter plates were washed three times with 0.05% PBST. Then, horseradish peroxidase (HRP)-conjugated anti-M13 monoclonal antibody (GE Healthcare) in 3% BSA/PBS was added into wells, and the plate was incubated for 1 h at 37 °C. After washing three times with PBST, 2,2′-azino-bis-3-ethylbenzothiazoline-6-sulfonic acid solution (Thermo Scientific) was used as the substrate for HRP. Absorbance was measured at 405 nm with a Multiskan Ascent microplate reader (Labsystems, Helsinki, Finland).

To select reactive clones from the randomized libraries, phage ELISA was performed as described previously [64] using recombinant MERS-CoV S RBD protein-coated microtiter plates. The nucleotide sequences of positive scFv clones were determined by Sanger sequencing (Cosmogenetech, Seoul, Korea).

### 4.5. Expression of scFv-hFc and IgG_1_

The genes encoding the selected scFv clones were cloned into a modified mammalian expression vector containing the hIgG_1_ Fc regions (hFc) at the C-terminus as described previously [67]. The expression vectors were transfected into HEK293F cells (Invitrogen), and the fusion proteins were purified by Protein A affinity chromatography as described previously [67].

For the expression of IgG_1_, genes encoding *V_H_* and *V_L_* were amplified from the phage clones, cloned into a mammalian expression vector, and transfected into HEK293F cells. Then, IgG_1_ was purified by Protein A affinity chromatography as described previously [68]. Then the eluate containing IgG_1_ was subjected to gel filtration chromatography. A total of 4 mg of IgG_1_ was injected at a flow rate of 1 mL/min and purified by gel filtration using a XK16/100 column packed with Superdex 200 pg at pH 7.4 (ÄKTA pure, GE Healthcare). The chromatogram was recorded at a UV absorbance of 280 nm. The fractions containing IgG_1_ were pooled by collection criteria and concentrated.

### 4.6. ELISA

Microtiter plates (Costar) were coated with 100 ng of recombinant S glycoprotein in coating buffer at 4 °C overnight. The wells were blocked with 3% BSA/PBS for 1 h at 37 °C. Both nebulized and non-nebulized scFv-hFc or IgG_1_ were serially diluted (5-fold, 12 dilutions starting from 500 nM for scFv-hFc fusion protein or 1000 nM for IgG_1_) in blocking buffer and added into individual wells. After incubation for 1 h at 37 °C, the microtiter plates were washed three times with 0.05% PBST. Then, HRP-conjugated rabbit anti-human IgG antibody (Invitrogen) in blocking buffer (1:5000) was added into wells, and the plate was incubated for 1 h at 37 °C. After washing three times with PBST, 2,2′-azino-bis-3-ethylbenzothiazoline-6-sulfonic acid solution (Thermo Scientific) was used as the substrate. Absorbance was measured at 405 nm using a microplate spectrophotometer (Multiskan GO; Thermo Scientific)

### 4.7. Nebulization

A nebulizer (Aerogen Pro, Aerogen, Galway, Ireland) was used for all experiments following the manufacturer’s instructions. The nebulizer containing 1 mL of scFv-hFc fusion proteins or IgG_1_ antibodies was placed on top of a 50-mL conical tube (SPL Life Sciences, Pocheon, Korea) and nebulized at a concentration of either 0.1, 0.3, or 1 mg/mL in PBS.

### 4.8. Microneutralization Assay

The virus (MERS-CoV/KOR/KNIH/002_05_2015, accession number KT029139.1) was obtained from the Korea National Institute of Health (kindly provided by Dr. Sung Soon Kim) and propagated in Vero cells (ATCC CCL-81) in Dulbecco’s Modified Eagle’s Medium (DMEM, Welgene, Gyeongsan, Republic of Korea) in the presence of 2% fetal bovine serum (Gibco). The cells were grown in T-75 flasks, inoculated with MERS-CoV, and incubated at 37 °C in a 5% CO_2_ environment. Then 3 days after inoculation, the viruses were harvested and stored at −80 °C. The virus titer was determined via a TCID_50_ assay [69].

A neutralization assay was performed as previously described [19]. Briefly, Vero cells were seeded in 96-well plates (1 × 10^4^ cells/well) in Opti-PRO SFM (Thermo Scientific) supplemented with 4 mM L-glutamine and 1× Antibiotics-Antimycotic (Thermo Scientific) and grown for 24 h at 37 °C in a 5% CO_2_ environment. Two-fold serially diluted scFv-hFc fusion proteins were mixed with 100 TCID_50_ of MERS-CoV, and the mixture was incubated for 30 min at 37 °C. Then, the mixture was added to the Vero cells in tetrad and incubated for 4 days at 37 °C in a 5% CO_2_ environment. The cytopathic effect (CPE) in each well was visualized following crystal violet staining 4 days post-infection. The IC_50_ values were calculated using the dose-response inhibition equation of GraphPad Prism 6 (GraphPad Software, La Jolla, CA, USA).

### 4.9. Flow Cytometry

The scFv-hFc fusion proteins (2000, 1000, 250, or 200 nM) were incubated either with 200 nM of the recombinant S glycoprotein fused with a polyhistidine tag at the C-terminus (Sino Biological Inc.) or without S protein in 50 μL of 1% (*w*/*v*) BSA in PBS containing 0.02% (*w*/*v*) sodium azide (FACS buffer) at 37 °C for 1 h. The m336 scFv-hFc and irrelevant scFv-hFc fusion proteins were used as positive and negative controls, respectively. Huh-7 cells (hDPP4^+^) were added into v-bottom 96-well plates (Corning, Corning, NY, USA) at a density of 3 × 10^5^ cells per well, and then, the mixture was added to the wells. After incubation at 37 °C for 1 h, cells were washed three times with FACS buffer and incubated with FITC-labeled rabbit anti-HIS Ab (Abcam, Cambridge, UK) at 37 °C for 1 h. Then, the cells were washed three times with FACS buffer, resuspended in 200 μL of PBS, and subjected to analysis by flow cytometry using a FACS Canto II instrument (BD Bioscience, San Jose, CA, USA). For each sample, 10,000 cells were assessed, and the data were analyzed using the FlowJo software (TreeStar, Ashland, OR, USA).

### 4.10. SE-HPLC

Non-nebulized and nebulized samples were analyzed using Waters e2695 HPLC system (Waters Corporation, Milford, MA, USA) equipped with a BioSuite high-resolution size-exclusion chromatography column (250 Å 7.5 mm × 300 mm). Each sample (10 μg) was injected at a flow rate of 1 mL/min. The mobile phase was PBS (pH 7.4), and UV detection was performed at 280 nm/220 nm. The sample tray and column holder were maintained at 4 and 30 °C, respectively, throughout data acquisition. The molecular weights corresponding to the antibody peaks were calculated using the Empower software (Waters Corporation).

### 4.11. DLS Assay

DLS experiments were performed using a Zetasizer Nano S (Malvern Panalytical Ltd., Malvern, UK) and a 633-nm/4-mW laser at a 173 ° detection angle as described previously [37]. Non-nebulized and nebulized samples were analyzed by performing three acquisitions per sample. PBS (pH 7.4) was used as the reference solvent. The results were evaluated with the Zetasizer software 7.02 (Malvern Panalytical Ltd.).

### 4.12. PRNT Assay

Vero cells were seeded in 12-well plates (3.5 × 10^5^ cells/well) in Opti-PRO SFM supplemented with 4 mM L-glutamine and 1× Antibiotics-Antimycotic (Thermo Scientific) and grown for 24 h at 37 °C in a 5% CO_2_ environment. IgG_1_ antibodies were serially diluted three-fold in Dulbecco’s PBS (Welgene) and mixed with an equal volume of culture media containing MERS-CoV/KOR/KNIH/002_05_2015 (100 pfu). After incubation for 1 h at 37 °C in a 5% CO_2_ environment, the virus-antibody mixture was added to the cells and maintained for 1 h at room temperature. The mixture was then removed, and the cells were overlaid with 1% agarose in DMEM. After incubation for 2 days at 37 °C in a 5% CO_2_ environment, the cells were washed with PBS and fixed for 24 h with 4% paraformaldehyde. The agarose overlay was removed, and the cell monolayer was gently washed with water to remove residual agarose. The cells were stained with 0.5% crystal violet solution, and the plaques were counted manually. The number of plaques was plotted as a function of IgG_1_ antibodies, and the concentration of IgG_1_ at which the number of plaques was reduced by 50% compared to that in the absence of IgG_1_ (PRNT_50_) was calculated using GraphPad Prism 6.

## Figures and Tables

**Table 1:** Size-exclusion high-performance liquid chromatography (SE-HPLC) and dynamic light scattering (DLS) analysis

Antibody	SE-HPLC (% Monomer/% Aggregates)		DLS (% Monomer ± SD/% Aggregates ± SD)	
	Pre-Nebulization	Post-Nebulization	Pre-Nebulization	Post-Nebulization
C-8	100.0/0	97.9/2.1	100.0 ± 0/0	78.4 ± 3.5/21.6 ± 3.5
C-8-2-4B-10D	100.0/0	100.0/0	99.2 ± 0.7/0.8 ± 0.7	98.6 ± 0.4/1.4 ± 0.4
m336	100.0/0	99.4/0.6	96.6 ± 0.6/3.4 ± 0.6	77.5 ± 2.3/22.5 ± 2.3

## Supplementary Materials

Supplementary materials can be found at https://www.mdpi.com/1422-0067/20/20/5073/s1.

## Abbreviations

MERS-CoV	Middle East respiratory syndrome coronavirus
DPP4	dipeptidyl peptidase 4
RBD	receptor-binding domain
i.v.	intravenous
mAb	monoclonal antibody
CDR	complementarity-determining region
scFv	single-chain variable fragment
PBMC	peripheral blood mononuclear cell
SE-HPLC	size-exclusion high-performance liquid chromatography
DLS	dynamic light scattering
PRNT	plaque reduction neutralization test
