# Supplementary material for: Generation of a Nebulizable CDR-Modified MERS-CoV Neutralizing Human Antibody
Source: Int J Mol Sci. 2019 Oct 12;20(20):5073. doi: 10.3390/ijms20205073 (PMC6829326; doi:10.3390/ijms20205073)
Supplement: Supplementary file 1 [file ijms-20-05073-s001.pdf]

Table S1. CPE inhibition by scFv clones

Clone	IC_{50} (μg/mL)
C-8	6.45
10	9.61
15	2.78
20	3.22
34	4.43
119	9.61
42	4.67
46	4.67
47	3.03
48	2.40
m336	3.71

Table S2. Degenerate codons used in the randomized libraries

The first library																	
	HCDR1									HCDR2							
Kabat number	H26	H27	H28	H29	H30	H31	H32	H33		H51	H52	H52A	H53	H54	H55	H56	H57
Amino acid	G	G	T	F	S	S	Y	A		I	I	P	F	F	G	T	A
Degenerate codon				KWK			KAK			RWK	RWK		KWK	KWK			
Amino acids encoded by the degenerate codon				D,E,F,LV,Y			D,E,Y			I,M,N,K,V,D,E	I,M,N,K,V,D,E		F,L,Y,V,D,E	F,L,Y,V,D,E			

The second library

	HCDR1								HCDR2							
Kabat number	H26	H27	H28	H29	H30	H31	H32	H33	H51	H52	H52A	H53	H54	H55	H56	H57
Amino acid	G	G	T	E	S	S	E	A	I	I	P	F	F	G	T	A
Degenerate codon	GRK	GRK	RMK		RRK	RRK		GMK						GRK	RMK	GMK
Amino acids encoded by the degenerate codon	G,D,E	G,D,E	T,N,K,A,D,E		S,R,K,N,G,D,E	S,R,K,N,G,D,E		A,D,E						G,D,E	T,N,K,A,D,E	A,D,E

The third library																							
	LCDR1											LCDR2			LCDR3								
Kabat number	L27	L27A	L27B	L27C	L27D	L27E	L28	L29	L30	L31	L32	L50	L51	L52	L89	L90	L91	L92	L93	L94	L95	L96	L97
Amino acid	Q	S	L	L	H	S	N	G	Y	N	Y	L	G	S	M	Q	A	L	Q	T	P	L	T
Degenerate codon			SWK	SWK					KAK		KAK	SWK			RWK			SWK				SWK	
Amino acids encoded by the degenerate codon			L,F,H,Q,V,D,E	L,F,H,Q,V,D,E					Y,D,E		Y,D,E	L,F,H,Q,V,D,E			M,I,K,N,V,D,E			L,F,H,Q,V,D,E				L,F,H,Q,V,D,E	

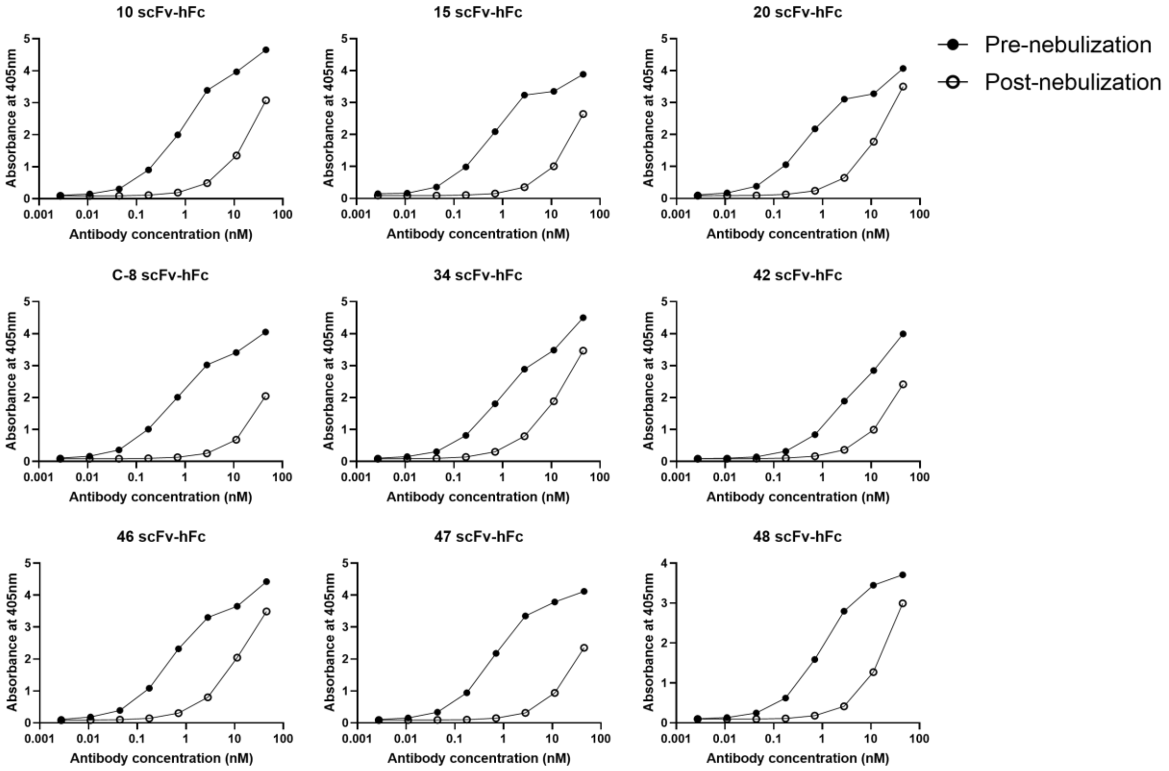

Figure S1. Reactivity of scFv clones before and after nebulization. The scFv-hFc fusion protein was nebulized at a concentration of 100 μg/mL, and aerosol was collected. After removing aggregated material by centrifugation, the supernatant and pre-nebulized scFv-hFc fusion proteins were subjected to ELISA using recombinant S glycoprotein-coated microtiter plates. The amount of bound scFv-hFc fusion protein was determined using HRP-conjugated anti-human IgG antibody and ABTS.

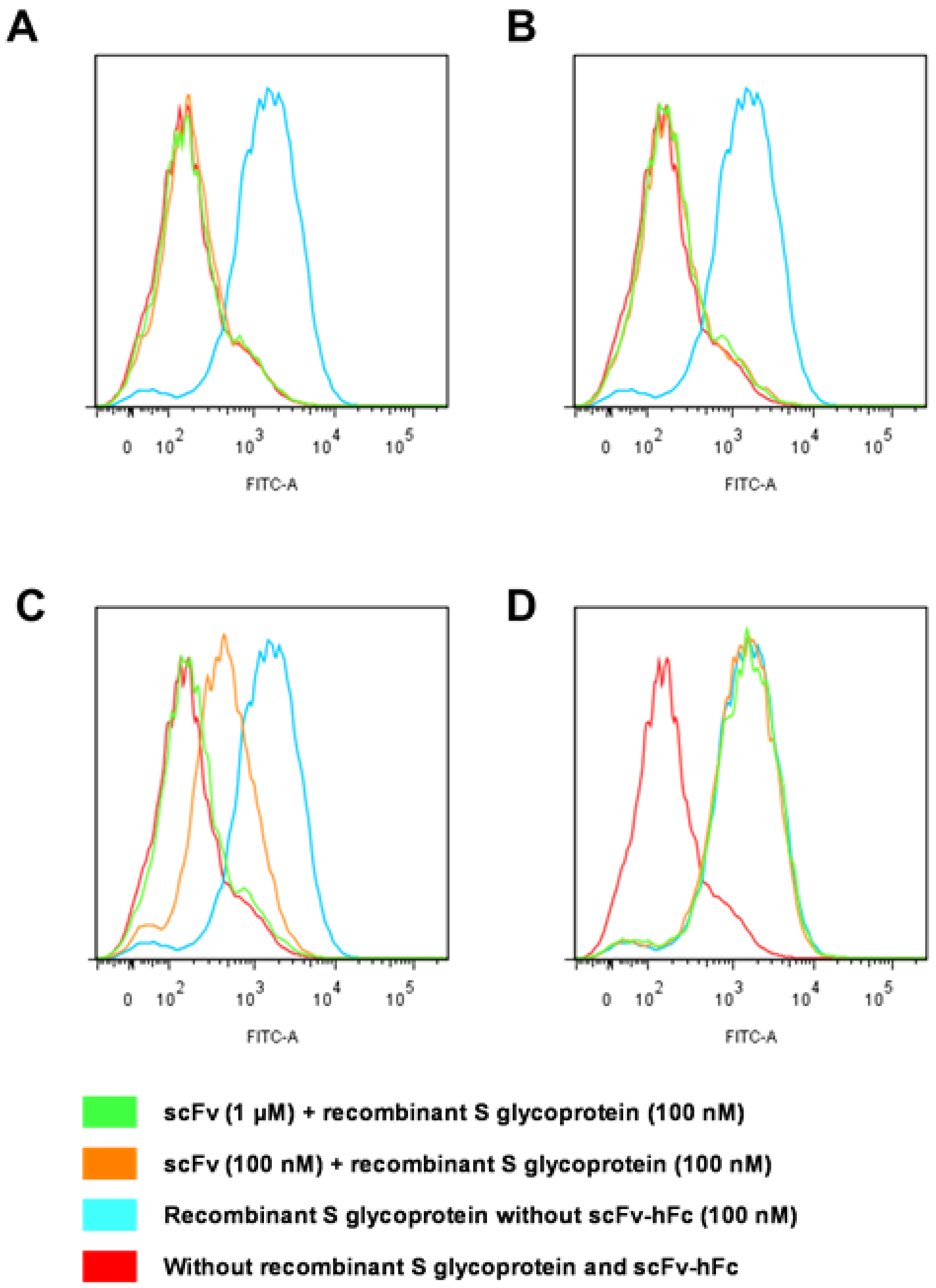

Figure S2. Flow cytometry analysis of the inhibition of recombinant S glycoprotein binding to hDPP4-expressing cells. C-8 (A), 48 (B), m336 (C), or negative control (D) scFv-hFc were mixed and incubated with recombinant S glycoprotein fused with a polyhistidine tag at the C-terminus. After incubation with Huh-7 (hDPP4+) cells, the relative amount of bound recombinant S glycoprotein was measured using FITC-conjugated anti-HIS antibody. Per each sample, 10,000 cells were monitored, and the data were analyzed using FlowJo software.

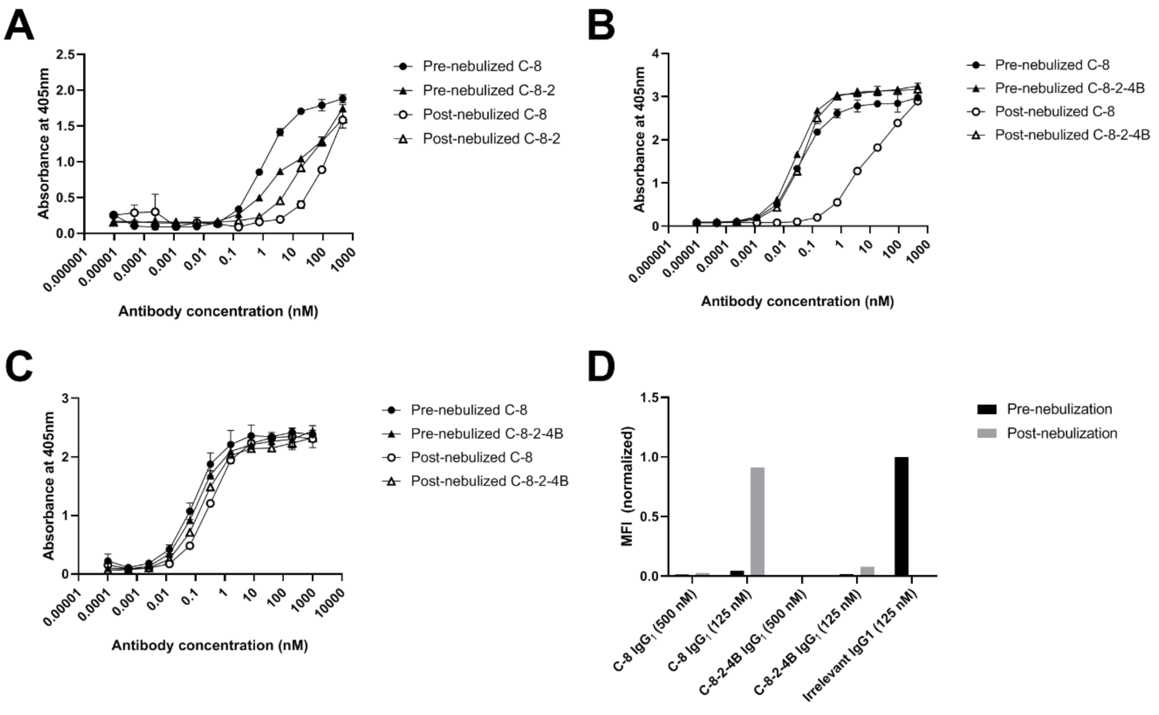

Figure S3. Biophysical characterization of C-8, C-8-2, and C-8-2-4B clones. Following nebulization at a concentration of 100 μg/mL or 300 μg/mL for scFv-hFc or IgG_1, respectively, aerosol was collected and subjected to ELISA (A–C) and flow cytometry (D). C-8-2 scFv-hFc (A), C-8-2-4B scFv-hFc (B), and C-8-2-4B IgG_1 (C) were serially diluted and incubated with recombinant S glycoprotein-coated microtiter plates. (D) C-8 IgG1 and C-8-2-4B IgG1 were incubated with recombinant S glycoprotein fused with a polyhistidine tag at the C-terminus, and the complex was allowed to react with hDPP4-expressing cells. The amount of bound recombinant S glycoprotein was measured using FITC-conjugated anti-HIS antibody. Data are representative of 10,000 cells for each sample.

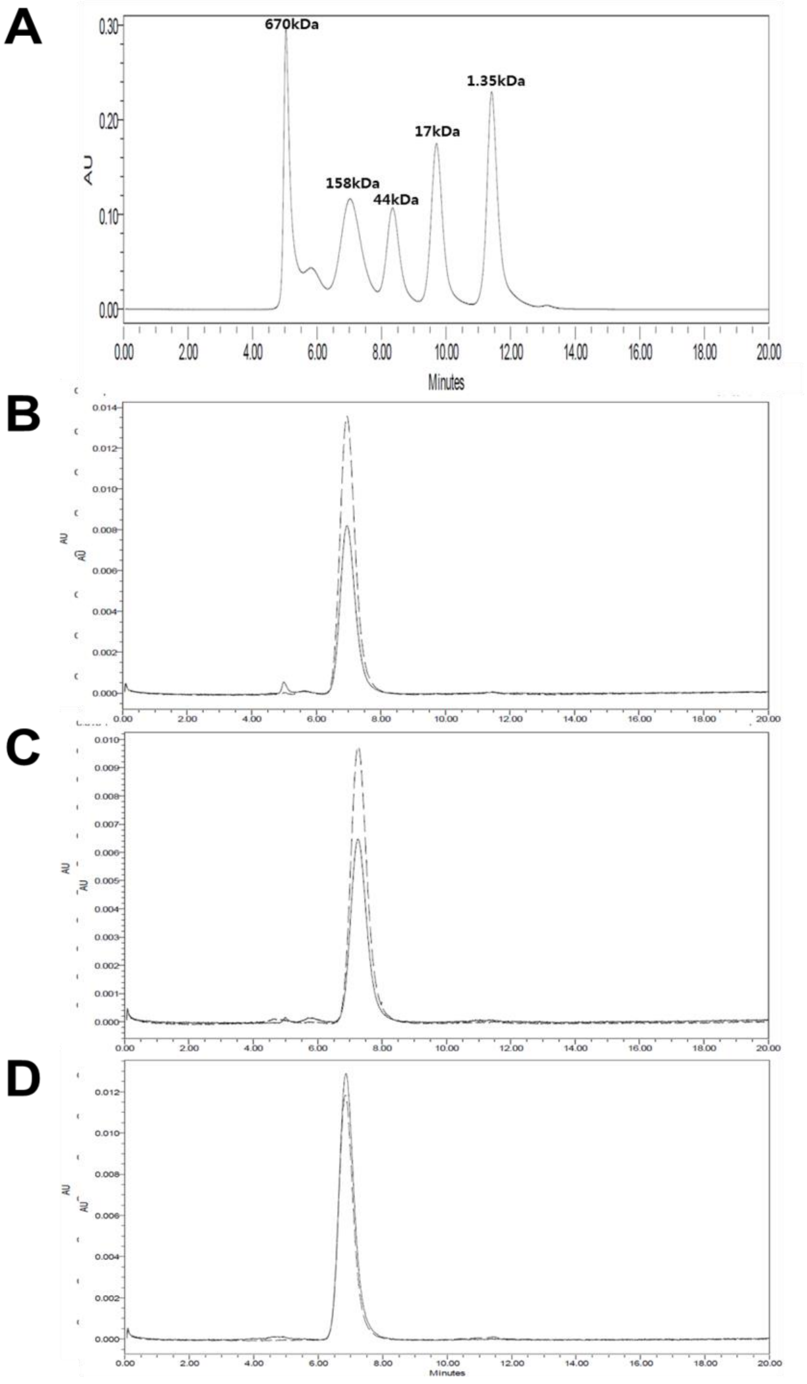

Figure S4. Size-exclusion chromatography of MERS-CoV IgG$_1$ antibody before and after nebulization. Pre-nebulized (dotted lines) and post-nebulized (solid lines) samples were analyzed using Waters e2695 HPLC system. Standard (A), C-8 IgG$_1$ (B) m336 IgG$_1$ (C), and C-8-2-4B-10D IgG$_1$ (D) were injected at a flow rate of 1 mL/min. The mobile phase was PBS (pH 7.4), and UV detection was performed at 280 nm. The molecular weights corresponding to the antibody peaks were calculated with Empower software.

Table S3. Primers used for the generation of the randomized libraries

**Primer set 1**	**Forward: 5′- AACTACGCACAGAAGTTCCAGGGCAG-3′**
	**Reverse: 5′-GGCCGGCCTGGCCTGAGGAGACGGTGACCGTG-3′**
**Primer set 2**	**Forward: 5′- GTGGCTCGGGCGGTGGTGGGGAGGTGCAGCTGGTGCAGTCTGG -3′**
	**Reverse: 5′-GGCCGGCCTGGCCTGAGGAGACGGTGACCGTG-3′**
**Primer set 3**	**Forward: 5′-GGCCCAGGCGGCCGAGCTCGTGATGACTCAGTCTCCA-3′**
	**Reverse: 5′-CCCACCACCGCCCGAGCCACCGCCACCAGAGGAGGAAGATCTAGAGGAACCACCTTTGATTTCCACCTTGGTCCCTC-3′**
